# A novel purification method of activated carbon-supported carbon nanotubes using a mixture of Ca(OH)_2_ and KOH as the ablation agent†

**DOI:** 10.1039/d0ra08346a

**Published:** 2021-01-04

**Authors:** Yongjie Hu, Linlin Zhang, Qixun Guo, Zhifeng Zheng, Yunquan Liu, Yueyuan Ye, Shuirong Li, Xingyong Jia, Duo Wang

**Affiliations:** Fujian Engineering and Research Center of Clean and High-valued Technologies for Biomass, College of Energy, Xiamen University Xiamen 361102 P. R. China duowang@xmu.edu.cn +86 5922188053 +86 5922188266; Graduate School of Chinese Academy of Agricultural Sciences Beijing 100081 China

## Abstract

Transition metals (Fe, Co, Ni) supported on activated carbons with different pore diameters (<2 nm, 10 nm, 50 nm) to synthesize carbon nanotubes (CNTS) are first investigated in this study. Through several characteristic analyses, Ni supported on 50 nm activated carbon is verified to be the most efficient catalyst among the samples for CNT growth. The optimum conditions for CNT growth are at a growth temperature of 750 °C with a reaction time of 45 min. Furthermore, a novel purification method for CNTs is proposed, in which KOH and Ca(OH)_2_ powder are pre-mixed with the crude CNTs and CO_2_ and N_2_ gas are introduced into this mixture. When KOH and Ca(OH)_2_ powder are used at a ratio of 2 : 1 under the atmosphere of CO_2_ and N_2_ at the temperature of 750 °C for 1 h, almost all of the amorphous carbon is ablated. Compared with KOH powder, the addition of Ca(OH)_2_ not only advances the ablation effect, but reduces the damage to CNTs.

## Abstract 2

Transition metals (Fe, Co, Ni) supported on activated carbons with different pore diameters (<2 nm, 10 nm, 50 nm) to synthesize carbon nanotubes (CNTS) are first investigated in this study.

## Graphical abstract

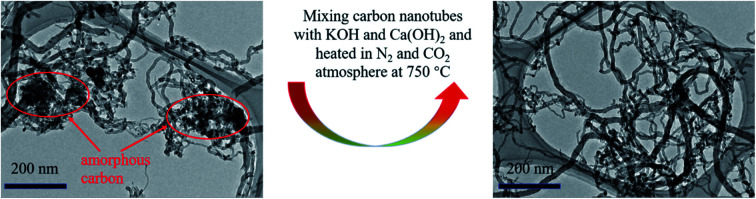

## 1. Introduction

Carbon nanotubes (CNTs) are regarded as one of the most promising materials due to their excellent mechanical and conductive properties, such as for electrode materials for fuel cells, lithium batteries and supercapacitors.^1–4^ The yield and purity of CNTs are important factors restricting their widespread industrial applications.^5^ Chemical vapor deposition (CVD) has become an effective method for preparing a large amount of CNTs due to its mild and controllable conditions.^6^ A series of active metals (such as Fe, Co and Ni, *etc.*) supported on porous materials (alumina, magnesia and silica, *etc.*) were studied for growing CNTs.^7–11^ The problem with the above-mentioned oxide support is that it increases the production cost of CNTs and requires subsequent pickling to remove them. More importantly, they often cannot be completely removed, afterwards, and the residual trace metals restrict the applications of CNTs in the biological field and part of the electrochemical field.^12^ Activated carbon as a relatively cheap and porous material can also be used as an efficient support for CNTs due to its abundant pores, ability to prevent the migration and agglomeration of metal atoms on its surface.^13^ In our previous study,^14^ activated carbon is verified to be a better nickel catalyst support than alumina for the growth of CNTs. Furthermore, the production of CNTs with activated carbon as the support will not encounter the problem of residual metal support in CNTs. However, the properties of activated carbon support and CNTs are similar, which makes it difficult to purify CNTs. An ideal purification and separation method of CNTs is worth developing and studying.

CNTs purification is extremely difficult due to the complexity of its impurity components, including metal particles, amorphous carbon, graphitized carbon and fullerene.^15^ Currently, CNTs purification methods mainly includes the physical separation (centrifugation, filtration, *etc.*), chemical oxidation, or a combination of these two methods.^16,17^ It is almost impossible to obtain high purity CNTs only using the physical method, thus it is often used as an auxiliary means in combination with a chemical separation.^18,19^ Many chemical oxidation methods for CNTs purification have been developed including liquid chemical oxidation, gas chemical oxidation and organic solvent. In a liquid oxidation method, sulfuric acid, nitric acid and hydrogen peroxide are generally used for removing the impurities from CNTs. The critical problems relate to the addition of some functional groups and meanwhile dramatically damage to CNTs.^17,20,21^ Some gases including Cl_2_, air, O_2_ and CO_2_ are also used as the oxygen agent for CNTs purification. Elaine *et al.*^22^ presented a purification technique for treating the CNTs with the highly reactive Cl_2_ gas at an temperature of 1000 °C for 10 min, and the content of metal impurities in CNTs less than 10 ppm. However, chlorine is not suitable for the elimination of carbonaceous impurities. Jeung *et al.*^23^ used chloroform (CHCl_3_) as a gas-phase purification approach to obtain metal-free MWCNTs, while it had no effect on carbon-based impurities. Nawal *et al.*^24^ reported a one-pot gas-phase treatment combining chlorine and oxygen. This method can remove both metallic and carbon impurities, however it also resulted in chlorine-containing functional groups present on the wall of CNTs. Furthermore, gas oxidation method tends to have a low CNTs yield in order to ensuring high purity.^25,26^

As an oxidizing gas, carbon dioxide can occur different degrees of oxidation reaction with amorphous carbon, CNTs, *etc.* Smith *et al.*^27^ verified the feasibility of selective oxidation of carbon-based impurities by carbon dioxide. It is generally known that the chemical agent KOH is very useful for the ablation of amorphous carbon.^28,29^ Furthermore, we accidentally found that the chemical agent Ca(OH)_2_ can improve the ablative capacity of KOH on amorphous carbon, which is better than their use alone. Considering this CNTs purification method has huge advantages that not only using non-toxic chemical agent in purification process, but feasibility in commercialization. Therefore, the green, non-toxic purification method of CNTs deserves to be further studied.

The objectives of this study were (1) to evaluate the effect of different transition metals supported on activated carbons with different pore size for CNTs growth, (2) to determine the operating parameters (*i.e.*, growth temperature, growth time, *etc.*) on CNTs yield and quality and (3) to examine the feasibility of using CO_2_, KOH and Ca(OH)_2_ as the purification agent of CNTs.

## 2. Material and methods

### 2.1. Material

The chemical reagents, Fe(NO_3_)_3_·9H_2_O (99.9%), Co(NO_3_)_2_·6H_2_O (99.9%), Ni(NO_3_)_2_·6H_2_O (99.9%), KOH (99.9%) and Ca(OH)_2_ (99.9%), were purchased from Macklin Chemical Reagent Co. (Shanghai, China). Ethanol was provided by Sinopharm Chemical Reagent Co. (Beijing, China). Microporous carbon with specific surface area 3200 m^2^ g^−1^ (average pore diameter < 2 nm, named as M1) was made by ourselves and two mesoporous carbon with specific surface area 1800 m^2^ g^−1^ and 600 m^2^ g^−1^ (pore diameter about 10 nm, named as M2; and 50 nm, named as M3) were purchased from XFNANO (Nanjing, China). Nitrogen (99.999%), argon (99.999%), hydrogen (99.999%), carbon dioxide (99.999%) and methane (99.999%) were all provided by Linde Industrial Gases.

### 2.2. Preparation method

#### 2.2.1. Preparation of activated carbon

Coconut shells were washed with deionized water and dried in an oven. The completely dried coconut shells were pulverized and ground into powders. Weighed 10 g of the above powder accurately, added 30 g of KOH activator, stirred well and placed them into the center of a quartz tube in a furnace. The heating program was set from room temperature to 750 °C at a rate of 10 °C min^−1^ in a nitrogen atmosphere at a constant temperature of 750 °C for 3 h, and then naturally cooled to room temperature. The collected powder after the reactions was washed by 10 wt% hydrochloric acid to remove metal impurities, then washed by deionized water until neutrality. The powder was completed dried in a constant temperature blast oven at 60 °C.

#### 2.2.2. Preparation of nickel catalyst

Weighed 0.25 g of nickel nitrate hexahydrate, dissolved in the appropriate amount of ethanol in a 100 mL beaker, sonicated for 15 min. Then, 0.5 g of the activated carbon was added into the beaker. The mixture was stirred at room temperature for 12 h, and stood for 6 h. Finally, the mixture was put in 60 °C constant temperature blast oven for 12 h.

#### 2.2.3. Preparation of CNTs

0.1 g of the nickel catalyst was added into a quartz tube, which was further placed in a vertical tube furnace. The temperature was raised from room temperature to 750 °C in an argon atmosphere at a flow rate 40 mL min^−1^. Then hydrogen as reducing gas was introduced at a flow rate 40 mL min^−1^. After 30 min, the carbon source gas methane was also introduced at a flow rate 10 mL min^−1^, meanwhile, the flow rate of hydrogen was reduced to 20 mL min^−1^. The switch valve of hydrogen and methane was turned off after 40 min of reaction, and the temperature was reduced to room temperature under an argon atmosphere.

#### 2.2.4. Purification of carbon nanotubes

**Fig. 1:**
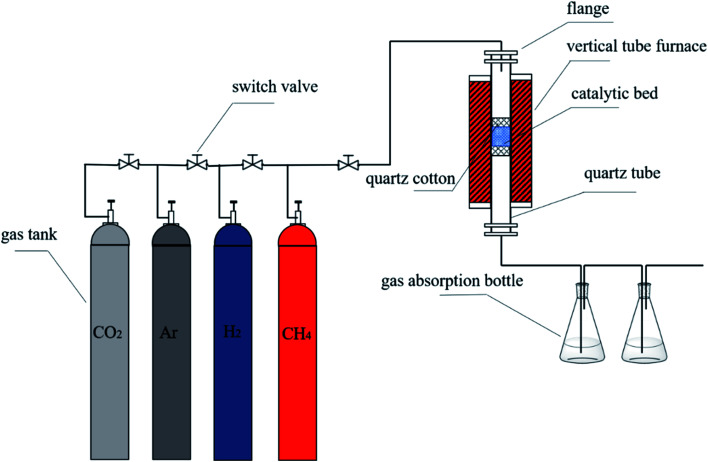
Schematic diagram of the experimental system.

The above raw product was immersed in concentrated hydrochloric acid, stirred at room temperature for 6 h, then washed with deionized water until neutral. When the raw product was completely dried, the mixture of raw product, KOH and Ca(OH)_2_ were placed in glass beaker in different molar ratio of 0 : 3, 1 : 2, 1 : 1, 2 : 0, 2 : 1 and 3 : 0, respectively. The mixture was uniformly mixed and placed in the quartz tube, the temperature was raised from room temperature to 750 °C with nitrogen at a flow rate of 200 mL min^−1^. Then, carbon dioxide gas was introduced at 100 mL min^−1^ with total gas flow rate 200 mL min^−1^ and for 1 h of reaction. When the temperature was cooled to room temperature, the product was taken out and washed repeatedly by 6 mol L^−1^ hydrochloric acid. Then, the product was further washed with deionized water until neutral and was completely dried in 60 °C constant temperature blast oven. The obtained CNTs were named PCNTs 1–6 according to different ratios of KOH and Ca(OH)_2_. The preparation and purification system were shown in Fig. 1.

### 2.3. Characterization method

Metal species and the crystal structure of catalysts were characterized by XRD patterns which were collected in a X-ray diffractometer (XRD, Ultima IV, Rigaku Co, Japan) with Cu Kα radiation at 40 kV and 35 mA. The start and end positions in the 2*θ* range from 10° to 90° with a scanning step of 0.002°. The morphology and quality of CNTs were monitored using a field emission scanning electron microscopy (FESEM; ZEISS Sigma 500 VP) and a transmission electron microscope (TEM, JEM-2100F JEOL, USA). Raman spectra were obtained using a TakRam Raman microscope with Raman shift from 200 to 4000 cm^−1^ at a wavelength of 514 nm. The thermal stability of CNTs before and after purification process was analyzed by a temperature-programmed oxidation (TPO, STA449F5 TGA, NETZSCH, Germany) under the atmosphere of air.

## 3. Results and discussion

### 3.1. Effect of different catalyst on CNTs growth

#### 3.1.1. XRD analysis of different catalyst

**Fig. 2:**
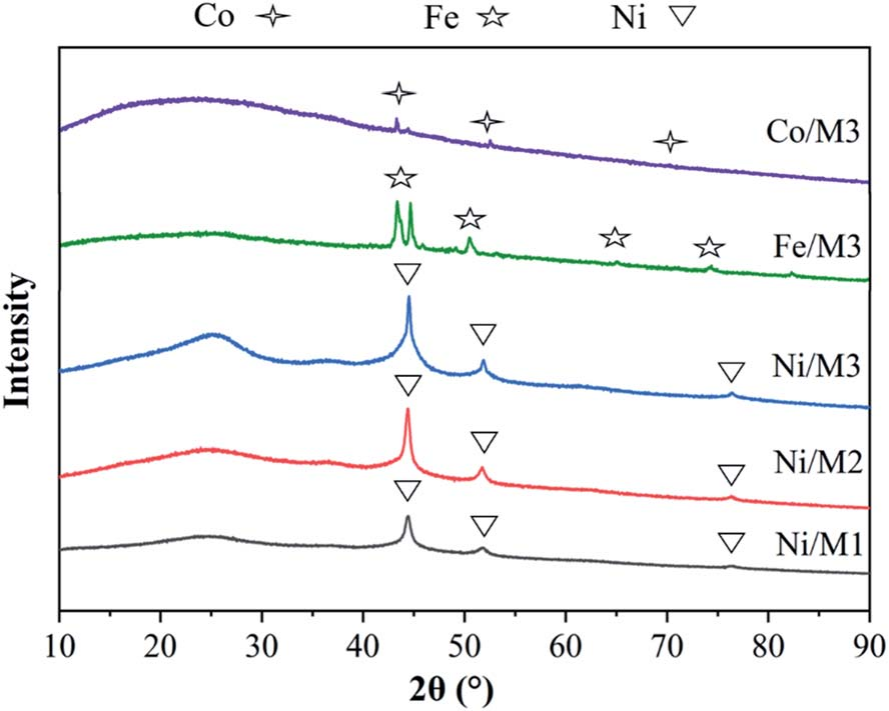
XRD analysis of different catalysts.

The XRD patterns of different catalysts were shown in Fig. 2. The main peaks of Co, Fe and Ni were respectively observed in Co/M3, Fe/M3 and Ni/M3 catalysts due to a sufficiently reduction of hydrogen. The same three sharp nickel diffraction peaks appeared at 44.2°, 51.8° and 76.4° attributed to Ni/M1, Ni/M2 and Ni/M3, indicating the presence of active nickel sites which was beneficial to the decomposition of CH_4_.^30^ According to the Scherrer formula, the crystallite size and half height width were closely related,^31^ thus the average crystallite size of Ni was calculated as 10.4, 14.8 and 18.04 nm approximately for Ni/M1, Ni/M2 and Ni/M3 respectively.

#### 3.1.2. SEM observation

**Fig. 3:**
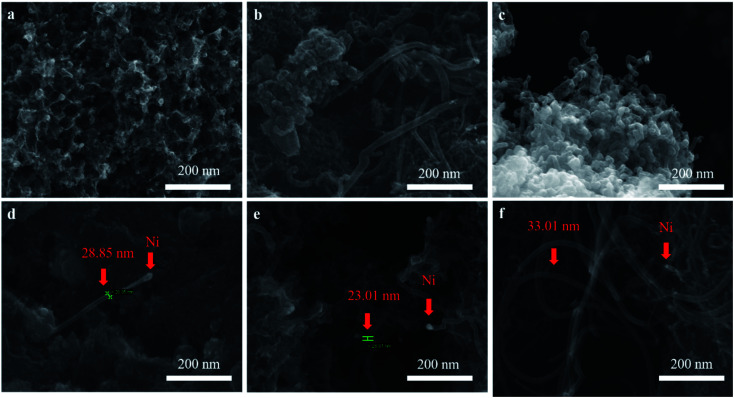
(a) SEM images of Ni/M3 catalyst, (b) CNTs synthesized by Fe/M3, (c) CNTs synthesized by Co/M3, (d) CNTs synthesized by Ni/M1, (e) CNTs synthesized by Ni/M2, (f) CNTs synthesized by Ni/M3.

The dispersion of nickel on M3 and the effect of different catalysts on CNTs growth was observed as shown in Fig. 3. As expect, nickel particles were dispersed uniformly on the surface of M3 (Fig. 3a). It can be observed that the amount of CNTs on Ni/M3 (Fig. 3f) was more than Fe/M3 (Fig. 3b) and Co/M3 (Fig. 3c), indicating Ni/M3 owned the best catalytic performance. Furthermore, the amount of CNTs on Ni/M3 was more than Ni/M1 (Fig. 3d) and Ni/M2 (Fig. 3e), indicating that larger pore was benefit for CNTs growth. Nickel particles were found that mainly exist on the top of CNTs, which was in accordance with the tip growth mechanism of CNTs.^32,33^ It also indicated that the same tip growth mechanism was suitable for the different catalyst supports including alumina, silica and activated carbon.^34,35^ Based on the tip growth theory, the weaker adsorption of nickel particles on the support could result in the better growth of CNTs.^6,10^ In this work, the adsorption of nickel nanoparticles on M3 could be the weakest one among the samples, advancing the better growth of CNTs. It can also be observed that the average diameter of CNTs on Ni/M3 was about 33.01 nm, which was larger than 28.85 nm of Ni/M1 and 23.01 nm of Ni/M2. According to Ateia *et al.*^36^ and Mionic *et al.*,^37^ the larger size of metal particles resulted in the growth of larger diameter CNTs. From the results of XRD analysis, the average size of nickel particles on M3 was 18.04 nm which was the largest one of these samples. Thus, it was easy to understand that the diameter of CNTs on Ni/M3 was the largest one.

#### 3.1.3. Effect of different catalysts on CNTs yield

**Fig. 4:**
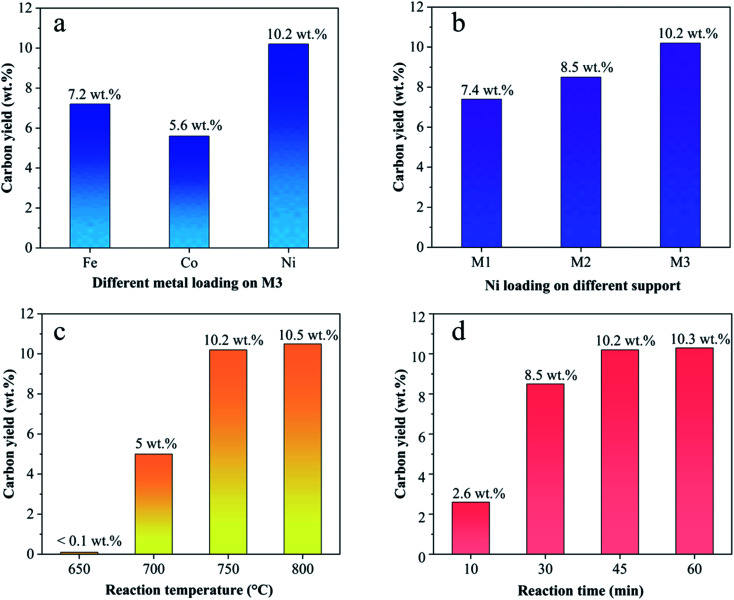
(a) Effect of Fe, Co and Ni loading on carbon yield, (b) effect of Ni loading supported by M1, M2 and M3 on carbon yield, (c) effect of reaction temperature on carbon yield using Ni/M3 catalyst, (d) effect of reaction time on carbon yield using Ni/M3 catalyst.

Fig. 4 shows the carbon yield of various catalysts for CH_4_ decomposition under different CNTs growth conditions. The carbon yield, *Y*_c_, is defined as 
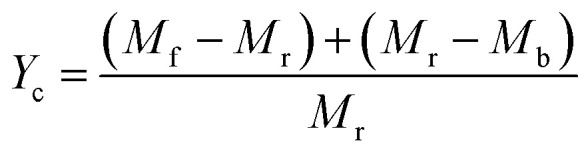
, where *M*_r_ and *M*_f_ corresponded to the weight of samples before and after the preparation process of CNTs, and *M*_b_ corresponded to the weight of samples without CH_4_ decomposition reaction under the same synthesis conditions. The carbon yield of different active metals including Fe/M3, Co/M3 and Ni/M3 was 7.2 wt%, 5.6 wt% and 10.2 wt%, respectively (Fig. 4a). Nickel catalyst exhibited better catalytic performance, which was consistent with the studies of Li *et al.*^14^ However, Kibria *et al.*^38^ reported that Fe owned better carbon yield than Ni while using alumina as support, which was due to that the carbon solubility of iron was higher than nickel. However, in this work, activated carbon was used as the catalyst support, which could also be a carbon source in CNTs growth process as reported by Hunley *et al.*^39^ Nickel particles were confirmed that it had a higher carbon diffusion rate than iron particles.^40^ It indicated that CNTs growth could be faster on nickel catalyst when enough carbon atoms, such as Ni/M3 catalyst, were appeared on the surface of metal catalyst. As shown in Fig. 4b, higher carbon yield of 10.2 wt% was obtained by Ni/M3, compared to 7.4 wt% of Ni/M1 and 8.5 wt% of Ni/M2. It indicated that the catalyst support with larger pore size was beneficial for CNTs growth.

The effect of reaction temperature on CNTs growth for Ni/M3 was shown in Fig. 4c when the reaction time was fixed at 45 min. When the reaction temperature was increased from 650 to 750 °C, the carbon yield significantly increased from 0.1 to 10.2 wt%. With increasing the reaction temperature to 800 °C, the carbon yield only slight increased to 10.5 wt%, indicating that further increasing temperature has little effect on the carbon yield. The effect of reaction time on CNTs growth was shown in Fig. 4d. The carbon yield was increased from 2.6 to 10.2 wt% with increasing the reaction time from 10 to 45 min. When the reaction time was further increased to 60 min, the carbon yield only slightly increased to 10.3 wt%. Higher reaction temperatures and longer reaction times require use of more heating energy and more CH_4_ gases, so the minimal but reasonably effective temperature and time are desired. Therefore, 750 °C and 45 min were chosen as an appropriate reaction temperature and time to test catalyst performance in other experiments.

### 3.2. Growth conditions of CNTs

#### 3.2.1. SEM observation

**Fig. 5:**
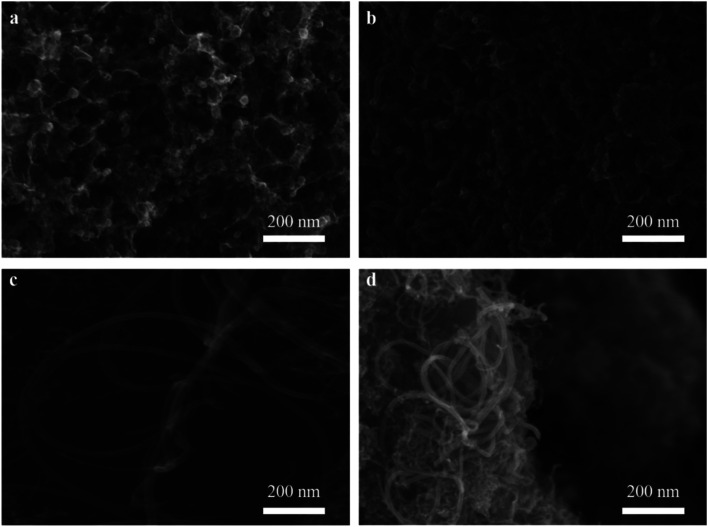
Effect of different reaction temperatures at 650 °C (a), 700 °C (b), 750 °C (c) and 800 °C (d) on CNTs quality.

The effect of different reaction temperatures on CNTs quality was observed by SEM (Fig. 5). At a relatively low temperature of 650 °C, the catalyst was well dispersed without obvious agglomeration, but almost no tubes were found on the surface of catalyst (Fig. 5a). At 700 °C, a large amount of CNTs with a diameter of 33.5 nm were observed, and a few defects were also found on their rough surface (Fig. 5b). It indicated that a relative low temperature resulted in poor crystalline structure and graphitization degree. When the temperature reached 750 °C, the length of CNTs increased significantly and its diameter slightly decreased to about 28 nm, meanwhile, the surface of CNTs became smooth (Fig. 5c). It indicated that higher temperature was benefit for higher quality CNTs with fewer defects. With increasing the temperature to 800 °C (Fig. 5d), the diameter of CNTs was distributed from less than 10 nm to 28 nm approximately, indicating that higher temperature tended to the growth of smaller diameter CNTs. The similar phenomenon was also reported by Ateia *et al.*^36^

**Fig. 6:**
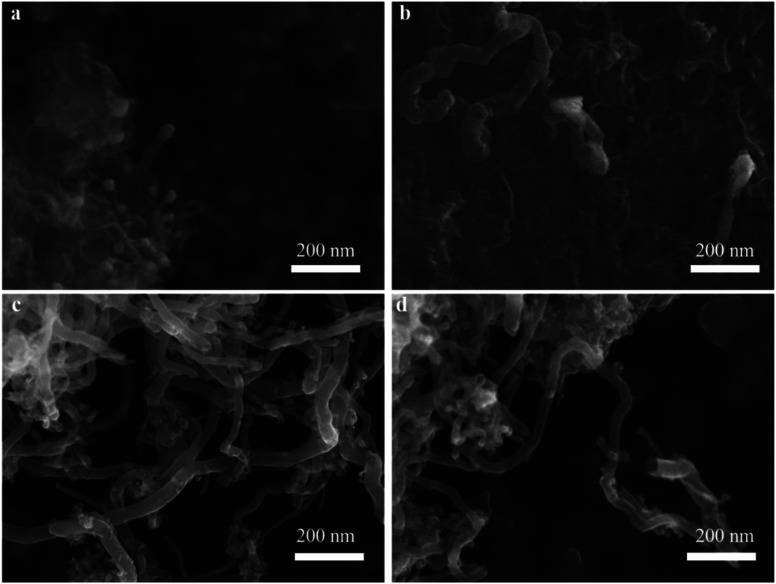
Effect of different time at 10 min (a), 30 min (b), 45 min (c) and 60 min (d) on CNTs quality.

The effect of different reaction times on CNTs quality was also observed by SEM (Fig. 6). When the reaction time was fixed at 10 min, the diameter of CNTs was about 23.23 nm, while the length of CNTs was still very short (Fig. 6a). With increasing the reaction time to 30 min (Fig. 6b), as expected, CNTs length gradually became longer, while these growing CNTS were stacked randomly and disorderly. When the reaction time reached 45 min (Fig. 6c), the diameter of CNTs increased to 32.55 nm and the length was also further increased. Furthermore, the density of CNTs on the catalyst surface also increased. With further increasing the reaction time to 60 min, it was interesting to note that the amount of CNTs decreased significantly even if carbon yield still increased as shown in Fig. 4d. The decreased amount of CNTs may due to the fact that CNTs could be etched into carbon fragments *via* H_2_.^41^

### 3.3. Purification effect on CNTs

#### 3.3.1. The ratio of KOH and Ca(OH)_2_

**Fig. 7:**
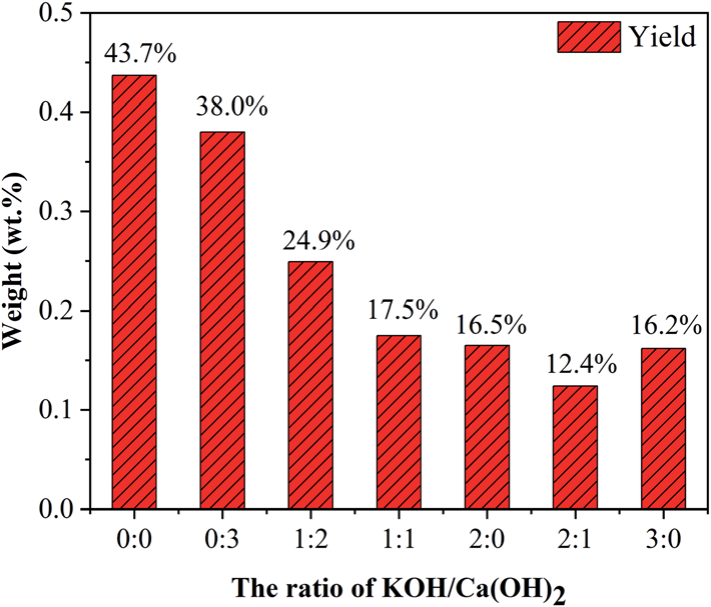
CNTs yield at different ratios of KOH and Ca(OH)_2_.

As shown in Fig. 7, with increasing the ratio of KOH/Ca(OH)_2_ from 0 : 0 to 3 : 0, CNTs yield gradually decreased. It indicated that the purity of obtained CNTs gradually increased. The purified yield 
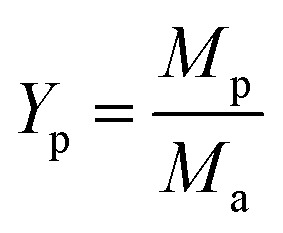
, where *M*_a_ and *M*_p_ were the weight of samples before and after purification process. It should be noted here that only about 10 wt% of our samples are CNTs. Therefore, the significance of purified yield just represents the ability to remove amorphous carbon. For PCNTs-CO_2_, the purified yield reached 43.7 wt% when only CO_2_ was introduced without KOH and Ca(OH)_2_. It indicated that lots of amorphous carbons were still existed in the sample. For 0 : 3 of KOH/Ca(OH)_2_ ratio (PCNTs-1), the purified yield slightly decreased to 38.0 wt%, indicating that only using Ca(OH)_2_ was not enough for the removal of amorphous carbon. When KOH was added and the ratio of KOH/Ca(OH)_2_ was 1 : 2 (PCNTs-2), the ablation effect was greatly enhanced, and the purified yield reached 24.9 wt%. It indicated that strongly ablative ability of KOH was a crucial factor for removing amorphous carbon. Compared 16.5 wt% yield for PCNTs-4, the lowest yield of 12.4 wt% was found for PCNTs-5. It indicated that Ca(OH)_2_ agent was very helpful for the ablation of the amorphous carbon from CNTs. It was interesting to note that purified yield suddenly increased to 16.2 wt% when the ratio of KOH/Ca(OH)_2_ reached 3 : 0 (PCNTs-6). It again verified that Ca(OH)_2_ agent played a key role in a CNTs purification process. It may due to that calcium additive acted as a deterrent to the potassium deactivation and thus promoted the gasification of amorphous carbon by CO_2_.^42^

#### 3.3.2. SEM and TEM observation

**Fig. 8:**
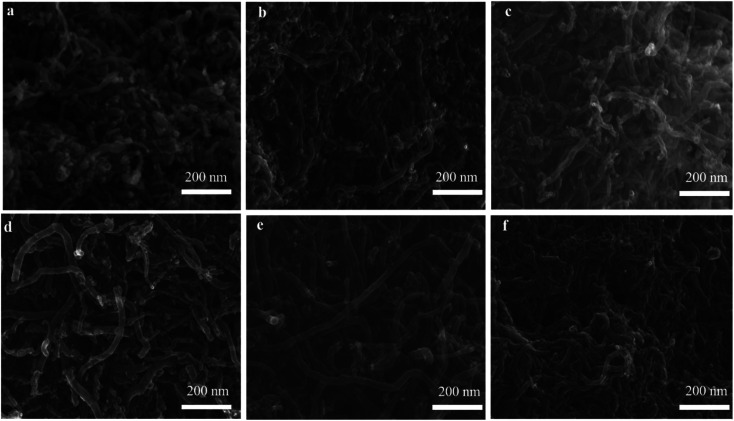
SEM images of PCNTs-CO_2_ (a), PCNTs-1 (b), PCNTs-2 (c), PCNTs-3 (d), PCNTs-5 (e) and PCNTs-6 (f).

**Fig. 9:**
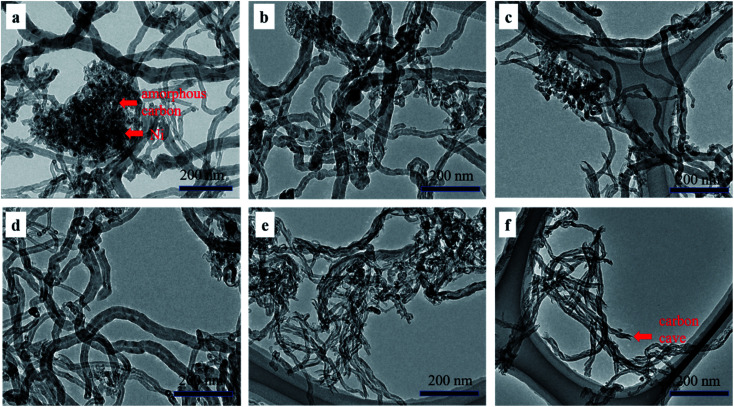
TEM images of PCNTs-CO_2_ (a), PCNTs-1 (b), PCNTs-2 (c), PCNTs-3 (d), PCNTs-5 (e) and PCNTs-6 (f).

The SEM and TEM images in Fig. 8 and 9 showed the morphology of CNTs after purification. As expected, a large amount of amorphous carbon could be seen in Fig. 8a and 9a due to the mild oxygen ability of CO_2_. After only mixing Ca(OH)_2_ with the raw CNTs, lots of amorphous carbon could also be seen in Fig. 8b and 9b, indicating the weak ablation ability of Ca(OH)_2_. When only KOH was mixed with the raw CNTs, amorphous carbons were almost eliminated completely as shown in Fig. 8f and 9f. Meanwhile, it can be clearly seen that the metal nanoparticles at the end of CNTs were also removed, leaving the carbon cavity and generating lots of defects on the wall of CNTs (Fig. 9f). It showed that the ablation effect of KOH was very strong, resulting in severely damage to the wall of CNTs. Compared with only using KOH, when both Ca(OH)_2_ and KOH was mixed with the raw CNTs, the appropriate ablation effect was obtained as shown in Fig. 8c–e and 9c–e. The amorphous carbon was basically eliminated from CNTs, indicating that the mixture of KOH and Ca(OH)_2_ is very effective in removing amorphous carbon and alleviate the damage of CNTs caused by KOH. The mechanism involved that potassium and calcium had synergistic effect to form a bimetallic carbonate, K_2_Ca(CO_3_)_2_, which showed higher reactivity for the gasification of amorphous carbon by CO_2_. The sharp peak of Raman spectra around 1077 cm^−1^ corresponds to K_2_Ca(CO_3_)_2_ (Fig. S1†).^43^ Furthermore, XRD pattern coincides well with the spectra of K_2_Ca(CO_3_)_2_ (Fig. S2†), indicating the existence of K_2_Ca(CO_3_)_2_ in the purification process.^44^ It was suggested that the bimetallic catalyst had a good distribution on the surface of particles due to its lower-temperature melting, thus improving the contact of potassium atom with amorphous carbon in the crude CNTs. Moreover, the bimetallic catalyst owned few catalytic effect for highly graphitized carbon such as CNTs.^44^

#### 3.3.3. Thermogravimetric analysis

**Fig. 10:**
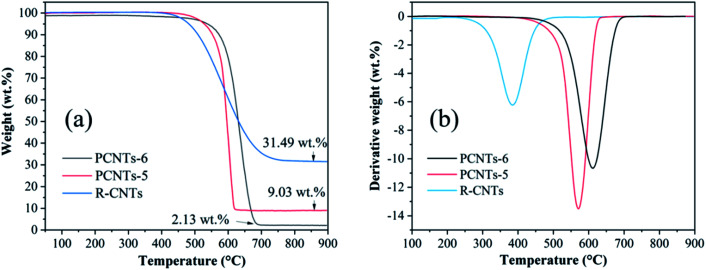
TGA (a) and DTG (b) analysis of R-CNTs, PCNTs-5 and PCNTs-6.

In order to compare the thermal stability and metal impurity content of CNTs before and after purification, thermogravimetric analysis was carried out. As shown in Fig. 10a, the weight loss of RCNTs, PCNTs-5 and PCNTs-6 began at around 390 °C, 480 °C and 530 °C, and three oxidation peaks were found at 394 °C, 570 °C and 610 °C, respectively (Fig. 10b). The weight loss at a relatively low oxidation temperature was related to amorphous carbon, while it was linked to the graphite carbon, such as CNTs, at higher oxidation temperatures due to its more stable characteristic.^45^ Compared with RCNTs, the oxidation temperature of PCNTs-5 and PCNTs-6 increased, indicating that most of amorphous carbon was removed during the purification process. The residual masses of R-CNTs, PCNTs-5 and PCNTs-6 was about 31.49 wt%, 9.03 wt% and 2.13 wt%, respectively. The residual mass was mainly composed of nickel oxide that converted by the metal nickel particles hidden in CNTs. The significant drop of residual mass after purification was attribute to the opening of CNTs ends during the purification process.

#### 3.3.4. Raman analysis

**Fig. 11:**
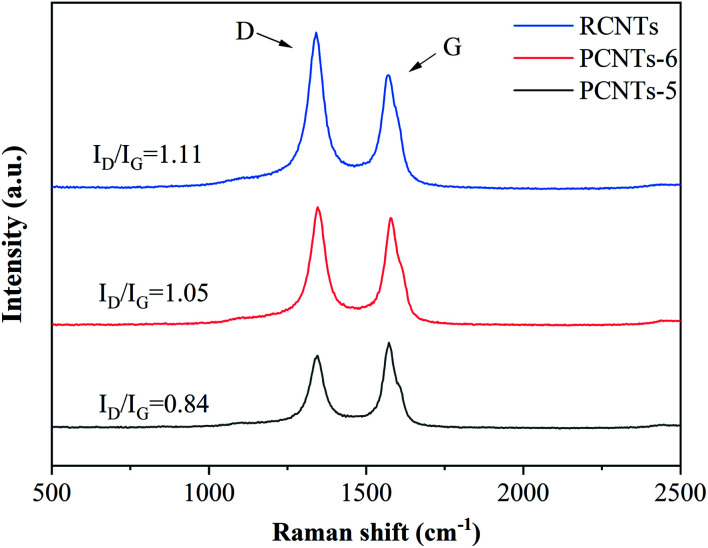
Raman spectra of CNTs before and after purification.

Raman was used to detect the quality of CNTs. The D-band (almost 1350 cm^−1^) is usually attributed to the amorphous or disordered carbon and represents the weakness of the activated bands in sp^2^ carbon materials. The G-band (nearly 1590 cm^−1^) is attributed to vibrations of sp^2^-bonded carbon atoms in graphite.^46^ The purification effect on raw CNTs was further studied by Raman analysis (Fig. 11). The ratio of *I*_D_/*I*_G_ was 1.11 for RCNTs, indicating that there was a large amount of amorphous carbon presented in the sample. The *I*_D_/*I*_G_ of PCNTs-5 reached 0.84, which was higher than 1.05 of PCNTs-6. It indicated that PCNTs-5 possess better crystal structure and graphitized structure. It again verified that the addition of Ca(OH)_2_ alleviated the strong ablation of KOH to decrease the defect intensity of CNTs, and meanwhile further enhanced the ability to remove amorphous carbon.

## 4. Conclusion

In this work, Fe, Co, Ni supported on activated carbons with three pore size (<2 nm, 10 nm, 50 nm) were prepared and used to produce CNTs. For the same activated carbon support, nickel catalyst is the most favorable for CNTs growth. When nickel was used as the active metal, 50 nm pore size activated carbon was better catalyst support based on the analysis of the yield and quality of CNTs. Based on the SEM, TEM and Raman analysis, the most suitable conditions for CNTs growth were at a growth temperature of 750 °C and a reaction time of 45 min.

Furthermore, a novel purification method for CNTs was proposed, in which KOH and Ca(OH)_2_ powder were pre-mixed with the crude CNTs and CO_2_ and N_2_ gas was introduced into this mixture. When KOH and Ca(OH)_2_ powder were used at a ratio of 2 : 1 under the atmosphere of CO_2_ and N_2_ at the temperature of 750 °C for 1 h, all the amorphous carbon were ablated thoroughly, the metal impurities content was 9.03 wt% after hydrochloric acid pickling. This study not only provides a novel method for the purification of CNTs, but generates the feasible routes for producing CNTs in industry.

## Conflicts of interest

There are no conflicts of interest to declare.

## Supplementary Material

RA-011-D0RA08346A-s001
