# Supplementary material for: A novel purification method of activated carbon-supported carbon nanotubes using a mixture of Ca(OH)2 and KOH as the ablation agent
Source: RSC Adv. 2021 Jan 4;11(2):1115–23. doi: 10.1039/d0ra08346a (PMC8693427; doi:10.1039/d0ra08346a)
Supplement: RA-011-D0RA08346A-s001 [file RA-011-D0RA08346A-s001.pdf]

Electronic Supplementary Material (ESI) for RSC Advances.
This journal is © The Royal Society of Chemistry 2020

Supporting Materials

A novel purification method of activated carbon-supported carbon nanotubes using the mixture of $Ca(OH)_2$ and KOH as the ablation agent

Yongjie Hu[a], Linlin Zhang[a], Qixun Guo[a], Zhifeng Zheng[a], Yunquan Liu[a], Yueyuan Ye[a], Shuirong Li[a], Xingyong Jia[b], Duo Wang[a,*]

a Fujian Engineering and Research Center of Clean and High-valued Technologies for Biomass, College of Energy, Xiamen University, Xiamen, P.R. China. 361102.

b Graduate School of Chinese Academy of Agricultural Sciences, Beijing, China. 100081.

*Corresponding author. Tel: +86 5922188266; Fax: +86 5922188053; E-mail address: duowang@xmu.edu.cn

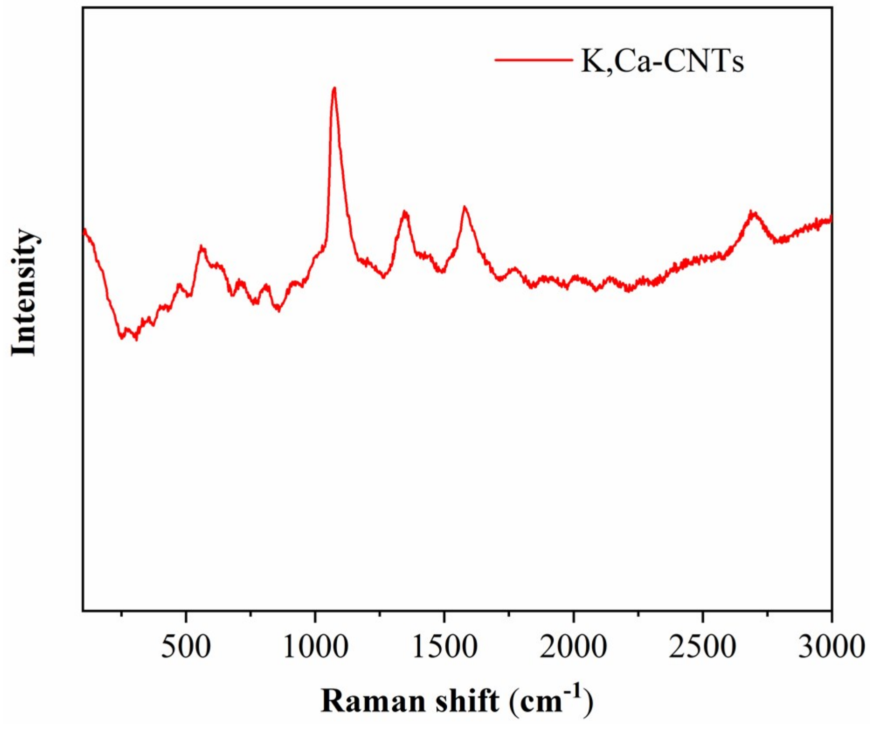

Figure S1. Raman spectra of K, Ca-CNTs.

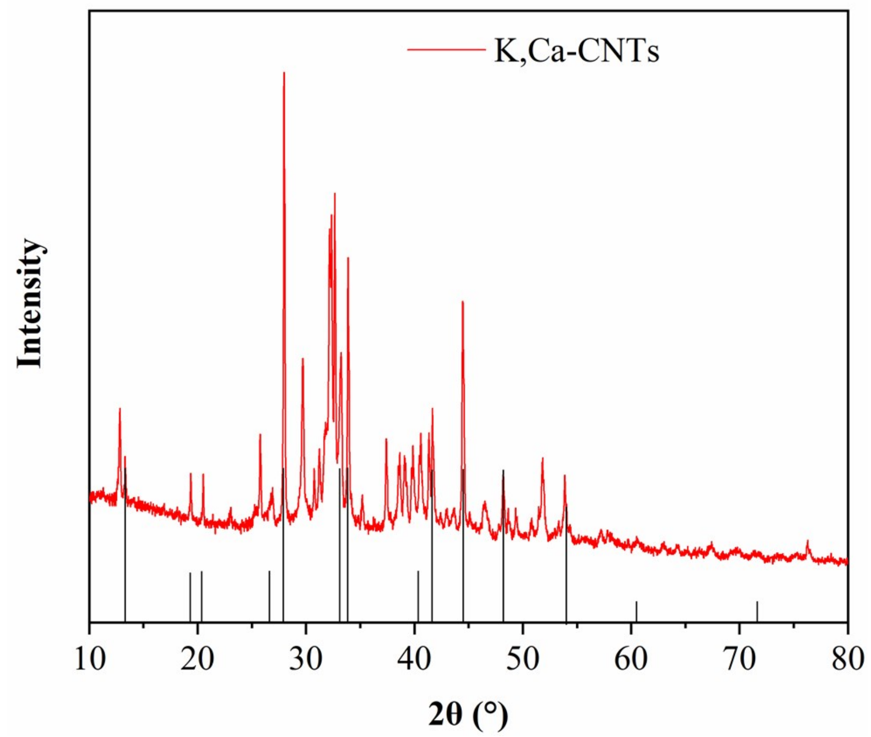

Figure S2. XRD spectra of K, Ca-CNTs.
